# From Audit to Action: Assessing and Implementing Improvements in the Operative Note Documentation for Day-Case Surgeries

**DOI:** 10.7759/cureus.97991

**Published:** 2025-11-28

**Authors:** Abdulwahab Mustafa, Monzir Salah Eldaw Mohammed Ahmed, Hashim Homaida, Islam Hamza Haroun Ahmedelamin, Mohamed Osman Mohamed Idres, Badawi ElAmin Ahmed ElKhalifa, Mohamedzain Ibrahim Omer Elfaki Elamin, Mohamedalhassan Osman Hassan Ahmed, Abdelhamid Abdelgayoum Suliman Bashir, Husam Eldin Abuelgassim Hassan Balila, Manal Hashim Saeid Othman, Eltayeb Elmamoun, Yusuf M. Y. Idris, Mohamed Alaeldin Mohamed Gasim, Hussein Jaafar Hussein Ahmed, Omer Abdelgader Waqeialla Ahmed, Rouida Elfadil Mohamed Ahmed Aboagla, Mohanad Elsafi Mossaad Elbashier, Malaz Siddeg Hamed Younis, Elwathig Abdalla

**Affiliations:** 1 General Surgery, Almanagil Teaching Hospital, Almanagil, SDN; 2 Vascular Surgery, Almanagil Teaching Hospital, Almanagil, SDN; 3 Urology, Almanagil Teaching Hospital, Almanagil, SDN; 4 General Surgery, Port Sudan Doctors Hospital, Port Sudan, SDN; 5 Medicine and Surgery, Almanagil Teaching Hospital, Almanagil, SDN; 6 Surgery, Almanagil Teaching Hospital, Almanagil, SDN; 7 Trauma and Orthopedics, Almanagil Teaching Hospital, Almanagil, SDN; 8 General Surgery and Vascular Surgery, Almanagil Teaching Hospital, Almanagil, SDN; 9 Emergency, Almanagil Teaching Hospital, Almanagil, SDN; 10 Orthopedics, Almanagil Teaching Hospital, Almanagil, SDN

**Keywords:** clinical audit day-case surgery, documentation compliance, operation note, quality of life, royal college of surgeons (rcs) standards

## Abstract

Background

Operative notes play an important role in ensuring patient safety, continuity of care, and medico-legal responsibility. Regardless of international standards, such as the Royal College of Surgeons (RCS) Good Surgical Practice, compliance is still low in most low- and middle-income countries, including Sudan.

Aim

The purpose of the closed-loop clinical audit is to assess and enhance adherence to the standards of operative note documentation in day-case surgeries at Almanagil Teaching Hospital, Almanagil, Sudan.

Methods

A quality improvement audit was performed over a period of three months, consisting of two cycles (pre- and post-intervention, with 53 operative notes each). A structured checklist was used to evaluate the documentation according to RCS standards. The interventions included staff education, standardized templates, and supervision. Chi-square tests were used to assess compliance between cycles.

Results

The compliance baseline was impaired in the main areas of estimated blood loss (0%), incision description (0%), deep vein thrombosis (DVT) prophylaxis (2%), and surgeon’s signature (8%). The most substantial change following the intervention was that almost all parameters improved (p < 0.001), with 100% compliance achieved in operative findings, complications, closure technique, DVT prophylaxis, and surgeon’s signature. There were continued inadequacies in the documentation of hospital file number (69.8%) and the reason why antibiotic prophylaxis was omitted (43.4%). Overall documentation increased by 41.3%, rising from 30.3% to 71.6% in Cycle 1 and Cycle 2, respectively.

Conclusion

The use of templates, education, and supervision as structured interventions made a significant contribution to enhancing the compliance of operative note documentation. Nevertheless, the lack of patient identifiers and the adequacy of the justification for antibiotic prophylaxis demonstrate that continuous audit cycles and e-documentation systems should be maintained to sustain the improvement.

## Introduction

Operative notes are essential components of surgical practice, serving as medical records, medico-legal documents, and tools for ensuring continuity of care. High-quality documentation promotes patient safety, facilitates postoperative management, and protects both surgeons and institutions. International standards - most notably the Royal College of Surgeons (RCS) of England’s Good Surgical Practice - outline core requirements such as patient identifiers, procedural details, intraoperative findings, complications, prophylaxis, and surgeon’s signature [1,2].

Despite these standards, numerous studies from both high- and low-income countries continue to report suboptimal operative note quality. Regional audits from Pakistan and India consistently show deficiencies in documenting operative findings, closure methods, and postoperative instructions [3-5]. Similar challenges are evident in Sudan, where audits from Dongola, Port Sudan, Elobeid, Wad Madani, and Kassala have demonstrated poor adherence to RCS guidelines, particularly concerning estimated blood loss, details of prostheses, postoperative advice, and surgeon’s signatures [6-12]. These gaps undermine patient safety, continuity of care, audit processes, and medico-legal accountability - issues made more significant in resource-limited settings.

Given these concerns, this audit aimed to evaluate adherence to RCS standards in operative note documentation for day-case surgeries at Almanagil Teaching Hospital, Almanagil, Sudan. The project sought to identify deficiencies, implement targeted interventions (education and standardized templates), and assess the impact through a closed-loop design.

## Materials and methods

This quality improvement project was conducted in the Department of Surgery of Almanagil Teaching Hospital, where the aim was to improve adherence to the required elements of operative note documentation during day-case surgeries. The project was implemented over a three-month period, from August 1st to October 31st, 2024, and consisted of two audit cycles, with an intervention introduced between them. The design followed a mixed retrospective and prospective observational approach, chosen because the retrospective review provided a baseline assessment of existing documentation practices, while the prospective review enabled evaluation of post-intervention performance in real time, ensuring that observed changes were attributable to the implemented strategies. Data collection in both cycles was carried out by two trained surgical residents, and, to minimize observer bias, both auditors underwent joint training and completed a pilot review of ten operative notes to ensure consistent interpretation of checklist items, achieving acceptable inter-rater agreement. Continuous process-improvement principles - including education, supervision, and structured documentation tools - supported the project throughout.

Tool and license

Documentation was assessed using the RCS Good Surgical Practice (2014) checklist, which is freely available for educational and audit purposes, and does not require special licensing [1]. All operative notes were selected using consecutive sampling, whereby every eligible day-case surgery performed during the audit periods was included to ensure completeness and avoid selection bias.

Pre-intervention (first cycle)

During the first audit cycle, 53 elective day-case surgery operative notes of the retrospective review were completed. The structured checklist, based on the RCS guidelines on Good Surgical Practice, was used to collect data. Every note was evaluated to record the necessary aspects, such as patient identifiers, date and time of operation, surgeon and assistants, operation procedure, findings, closing information, complications, postoperative guidance, and the signature of the concerned surgeon. Baseline results also revealed a lack of compliance in various areas, with the worst compliance observed in the documentation of estimated blood loss, details of the prosthesis, method of closure, and use of prophylaxis.

Intervention phase

**Figure 1:**
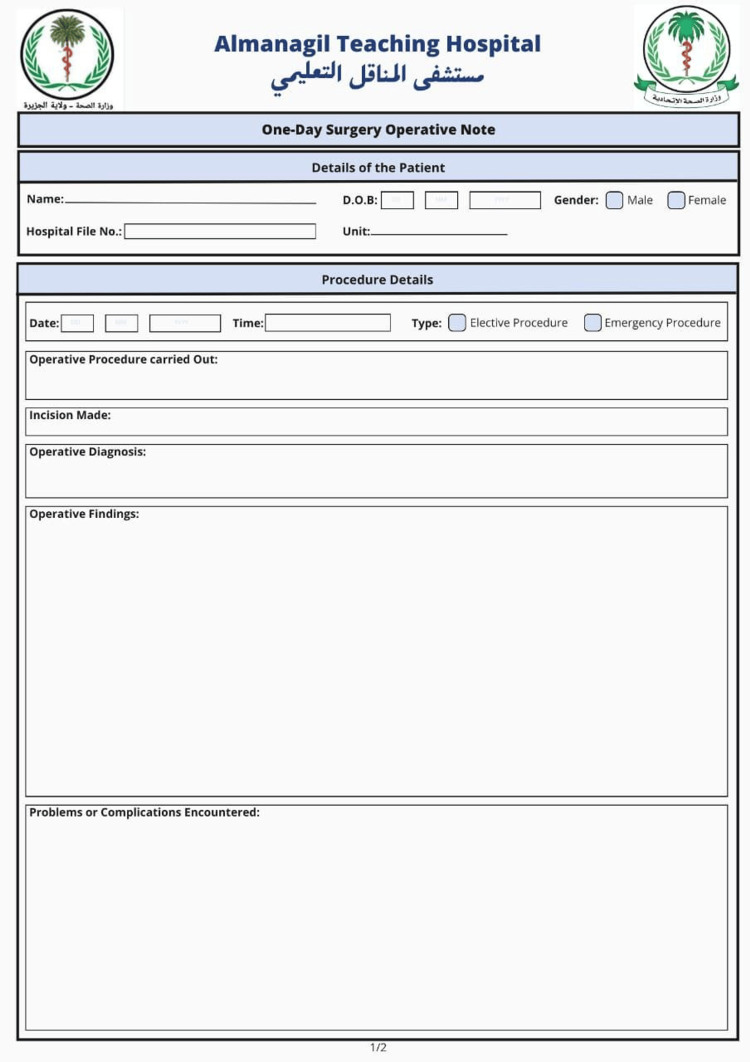
Front page of the newly designed operative note template used post-intervention

**Figure 2:**
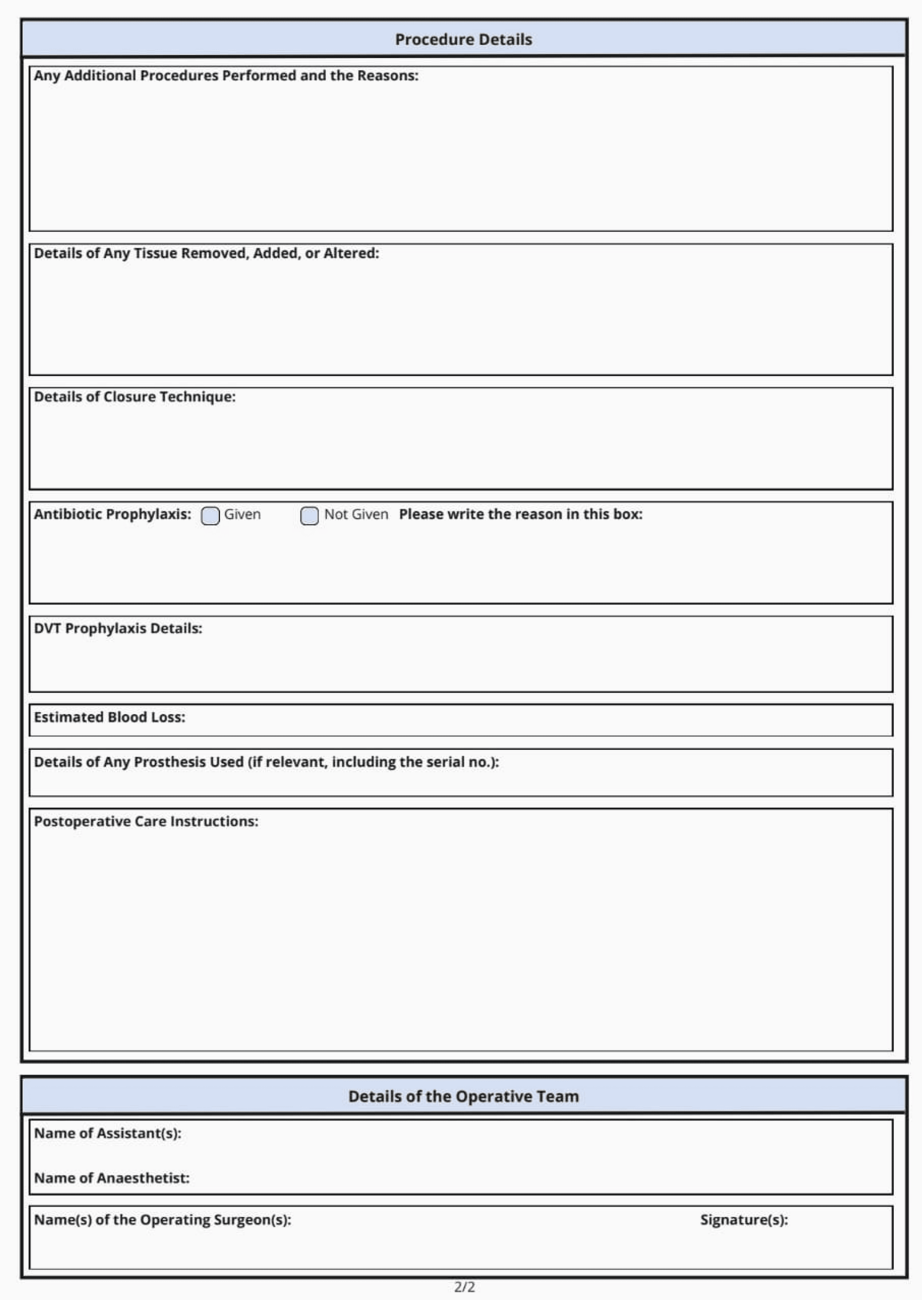
Back page of the new surgical operative note - procedure

Two weeks of intervention were applied to address these deficiencies. This intervention included organized educational activities among the surgical personnel, implementation of standardized templates of operative notes so that all the required elements were present, and placement of visual aids - including checklists and posters - in operating rooms. The first-cycle feedback was shared with the whole surgical team, and senior surgeons were advised to ensure close supervision of the junior staff to promote best-practice standards. Figure 1 shows the front page of the newly designed operative note template, while Figure 2 illustrates the back page.

Post-intervention (second cycle)

A second cycle of 53 operative notes was then prospectively checked after the intervention. The compliance was re-assessed using the same checklist. This later cycle showed significant progress in the records of operative diagnosis, intra-operative findings, pre-operative care, type of incision, method of closure, and complications. However, some areas - namely, the documentation of reasons why antibiotic prophylaxis was not administered and the recording of the hospital file numbers - still showed low compliance.

Data analysis

All operative notes from both cycles were evaluated against the RCS Good Surgical Practice documentation standards. Descriptive statistics summarized cycle-to-cycle performance, and Chi-square (χ²) tests (two-tailed, significance set at p < 0.05) were used to assess differences in compliance between cycles. Missing data were handled by classifying any unrecorded parameter as “not documented,” and these were included in the total denominator when calculating compliance percentages; no statistical imputation was used. Compliance was classified as satisfactory (≥90%), requiring improvement (70%-89%), or poor (<70%). Temporal trends across parameters were reviewed to quantify the impact of the intervention and to inform recommendations for sustaining improvements.

Ethical considerations

The audit committee of the hospital was approached to provide ethical approval. The data of all patients were anonymized to protect confidentiality, and the audit was performed according to the standards of the institution concerning quality improvement and clinical governance.

## Results

A total of 50 operative notes were reviewed during the first audit cycle, and 53 during the re-audit cycle following the implementation of the new structured operative note template and staff awareness sessions. The overall compliance rate increased substantially, from 31.6% in the first cycle to 92.4% in the second cycle, representing a 60.8-percentage-point improvement. This difference was statistically significant (χ² = 214.7, p < 0.001), indicating a marked enhancement in documentation completeness following the intervention. The completeness of documentation substantially improved across nearly all assessed parameters in the second cycle (Table 1).

**Table 1:** Comparison of adherence to surgical operative note standards in day-care surgeries

Parameter	First Cycle	Second Cycle	Absolute Improvement (% points)	χ² (df = 1)	p-value	Interpretation
Patient's full name	86.0 (43/50)	86.8 (46/53)	+0.8	0.00	1.000	Not significant
Date of birth	54.0 (27/50)	98.1 (52/53)	+44.1	42.6	<0.001	Significant improvement
Gender	34.0 (17/50)	98.1 (52/53)	+64.1	52.1	<0.001	Significant improvement
Hospital file number	2.0 (1/50)	69.8 (37/53)	+67.8	62.4	<0.001	Significant improvement
Unit	62.0 (31/50)	94.3 (50/53)	+32.3	13.9	<0.001	Significant improvement
Date of procedure	32.0 (16/50)	100.0 (53/53)	+68.0	65.2	<0.001	Significant improvement
Time of procedure	16.0 (8/50)	96.2 (51/53)	+80.2	70.3	<0.001	Significant improvement
Elective/emergency classification	0.0 (0/50)	98.1 (52/53)	+98.1	92.7	<0.001	Significant improvement
Operative procedure carried out	4.0 (2/50)	73.6 (39/53)	+69.6	55.4	<0.001	Significant improvement
Incision made	0.0 (0/50)	94.3 (50/53)	+94.3	87.1	<0.001	Significant improvement
Operative findings	12.0 (6/50)	98.1 (52/53)	+86.1	79.6	<0.001	Significant improvement
Operative diagnosis	50.0 (25/50)	100.0 (53/53)	+50.0	30.2	<0.001	Significant improvement
Problems/complications	2.0 (1/50)	100.0 (53/53)	+98.0	97.8	<0.001	Significant improvement
Extra procedures/reasons	6.0 (3/50)	100.0 (53/53)	+94.0	89.4	<0.001	Significant improvement
Tissue removed	2.0 (1/50)	100.0 (53/53)	+98.0	97.8	<0.001	Significant improvement
Closure technique	0.0 (0/50)	100.0 (53/53)	+100.0	98.5	<0.001	Significant improvement
Antibiotic prophylaxis	60.0 (30/50)	100.0 (53/53)	+40.0	25.6	<0.001	Significant improvement
Reason antibiotics not given	14.0 (7/50)	43.4 (23/53)	+29.4	9.6	0.002	Significant improvement
Deep vein thrombosis (DVT) prophylaxis	2.0 (1/50)	100.0 (53/53)	+98.0	97.8	<0.001	Significant improvement
Estimated blood loss	0.0 (0/50)	96.2 (51/53)	+96.2	90.3	<0.001	Significant improvement
Prosthesis details	2.0 (1/50)	100.0 (53/53)	+98.0	97.8	<0.001	Significant improvement
Post-op care instructions	46.0 (23/50)	100.0 (53/53)	+54.0	54.1	<0.001	Significant improvement
Assistant’s name(s)	2.0 (1/50)	96.2 (51/53)	+94.2	89.7	<0.001	Significant improvement
Anaesthetist’s name	0.0 (0/50)	96.2 (51/53)	+96.2	90.0	<0.001	Significant improvement
Surgeon’s name(s)	14.0 (7/50)	100.0 (53/53)	+86.0	78.9	<0.001	Significant improvement
Surgeon’s signature	8.0 (4/50)	100.0 (53/53)	+92.0	89.2	<0.001	Significant improvement

Patient demographic information

Documentation of patient details showed enhanced compliance. Entries for date of birth improved from 54.0% to 98.1%, gender from 34.0% to 98.1%, and hospital file number from 2.0% to 69.8%, all showing statistically significant improvement (p = 0.0). Patient's full name was already well-documented in the first cycle (86.0%) and remained stable (86.8%), with no significant difference (p = 1.0).

Operative and administrative details

There was a remarkable improvement in the documentation of operative scheduling and classification parameters. Date of procedure increased from 32.0% to 100.0%, time of procedure from 16.0% to 96.2%, and elective/emergency classification from 0.0% to 98.1% (p = 0.0 for all). Documentation of the unit responsible rose from 62.0% to 94.3% (p = 0.0002).

Operative description and findings

Core surgical details showed significant gains in completeness. Recording of the operative procedure carried out increased from 4.0% to 73.6%, incision made from 0.0% to 94.3%, and operative findings from 12.0% to 98.1%. Similarly, operative diagnosis rose from 50.0% to 100.0%. The documentation of problems or complications encountered improved from 2.0% to 100.0%. All these improvements were statistically significant (p = 0.0).

Surgical interventions and techniques

The inclusion of technical details saw major enhancements. Documentation of extra procedures or reasons, tissue removed, and closure technique all rose to 100% in the re-audit (from 6.0%, 2.0%, and 0.0%, respectively). Estimated blood loss and prosthesis details also improved markedly, from 0%-2% to over 96% and 100%. All demonstrated statistically significant improvement (p = 0.0).

Perioperative prophylaxis

Compliance with perioperative prophylaxis documentation increased notably. Antibiotic prophylaxis improved from 60.0% to 100.0%, while documentation of the reason antibiotics were not given improved from 14.0% to 43.4% (p = 0.0022). Deep vein thrombosis (DVT) prophylaxis recording rose from 2.0% to 100.0% (p = 0.0).

Postoperative and staff identification details

Documentation of postoperative care instructions improved from 46.0% to 100.0% (p = 0.0). Recording of key staff names also improved dramatically: assistant’s name(s) from 2.0% to 96.2%, anesthetist’s name from 0.0% to 96.2%, surgeon’s name(s) from 14.0% to 100%, and surgeon’s signature from 8.0% to 100% (p = 0.0 for all).

Overall improvement

Overall, 26 of the 27 audited parameters demonstrated statistically significant improvement following the intervention. The single parameter without significant change was the patient's full name, which was already consistently well-documented in the first cycle. The most substantial gains were observed in parameters that were initially poorly documented, such as elective/emergency classification, closure technique, assistant’s name, and prophylaxis details, each showing over a 90% increase in compliance.

## Discussion

The quality of operative note documentation at Almanagil Teaching Hospital improved significantly following the targeted interventions introduced through this closed-loop audit. The structured template, focused staff training, and increased supervision collectively enhanced compliance with the RCS standards, transforming documentation quality from very poor completeness in Cycle 1 to near-complete performance in Cycle 2. This transition demonstrates the powerful role of standardization and system-level redesign in producing consistent, reliable operative records.

These findings mirror those of other closed-loop audits conducted internationally. Improvements similar to those seen in this project were noted in studies from Pakistan and India, where standardized proformas and staff training substantially improved documentation, particularly regarding operative diagnosis, intraoperative findings, and closure details [3-6]. Regional experience from Dongola, Port Sudan, Elobeid, Wad Madani, and Kassala further supports the importance of structured interventions, although recurring issues - such as missing hospital file numbers and inadequate recording of antibiotic justification - remain common themes [7-12]. Synthesizing these findings indicates that documentation barriers are systemic across diverse resource settings and that structured tools, education, and repeated audit cycles are consistently effective solutions.

The most dramatic improvements were observed in parameters that were entirely absent in Cycle 1 - such as incision description, estimated blood loss, closure technique, DVT prophylaxis, and surgeon signature - all of which reached 100% compliance after the intervention. This suggests that the intervention’s success was not driven by the template alone; rather, it emerged from the synergy between the template (the “what”), education (the “why”), and senior supervision (the “how”). Without education to explain the clinical, administrative, and medico-legal importance of each data field - and without senior oversight ensuring adoption - the template alone would likely have produced far smaller gains.

Despite the overall success, some parameters remained below optimal compliance. Documentation of the justification for omitting antibiotic prophylaxis improved only to 43.4% in Cycle 2. This persistent weakness likely reflects workflow realities, including time pressure in preoperative preparation, variable awareness of prophylaxis guidelines, and the lack of a mandatory field prompting clinicians to document justification. Similar deficiencies have also been reported consistently across Sudanese and international audits [8-12], indicating that this issue may require stricter institutional policy, integration into surgical safety checklists, or the adoption of electronic systems with mandatory fields.

Likewise, despite improvements, documentation of the hospital file number remained below satisfactory levels (69.8%). Patient identifiers are central to continuity of care, legal protection, and safe health information management [1,2]. In settings without electronic medical records, reliance on manual data entry increases the likelihood of omissions. Simple, resource-appropriate strategies - such as barcode-based identifiers, administrative cross-checks, or sticky-label systems - could significantly reduce these errors.

The broader implications for low-resource healthcare settings are important. The interventions used - printed templates, brief training, and departmental supervision - are low-cost, require minimal infrastructure, and can be rapidly deployed in other district hospitals. The marked improvements achieved with such modest resources demonstrate that high-quality documentation is not restricted to technologically advanced systems, but can be attained through structured processes, repetition, and accountability.

To ensure the sustainability of these improvements, the Department of Surgery has established a forward-looking plan that embeds documentation quality into routine clinical practice. The department intends to conduct regular re-audits every 6-12 months to assess ongoing adherence, and a senior surgical registrar has been designated as the operative documentation lead to oversee compliance and address emerging gaps. Operative note training will be incorporated into the induction programs for new interns and residents to ensure early and consistent familiarity with the standardized template. Moreover, monthly random spot-checks of operative notes, combined with constructive feedback to the surgical team, will provide continuous monitoring and reinforcement. These measures collectively aim to cultivate a sustained culture of high-quality documentation and to safeguard the long-term continuity of the improvements achieved.

Several limitations must be acknowledged. The sample size was relatively small (50 and 53 cases), handwritten notes were reviewed manually (introducing observer bias), and the audit was limited to a single institution and to day-case surgeries. Sustainability beyond Cycle 2 has yet to be evaluated, and the absence of electronic documentation limited the ability to enforce mandatory fields - particularly for parameters that remained suboptimal. It is also possible that some of the observed improvements were influenced by the Hawthorne effect, whereby staff modified their behavior simply because they were aware that documentation practices were being monitored. However, experience from Atbara and other Sudanese institutions using similar RCS-based templates has also demonstrated substantial improvements in documentation quality, supporting the replicability of this model across comparable settings [13-15].

## Conclusions

This audit demonstrated that structured interventions - including standardized templates, staff education, and enhanced supervision - substantially improved adherence to operative note documentation standards at Almanagil Teaching Hospital. Overall documentation completeness improved from 41.3% in Cycle 1 to 71.6% in Cycle 2, with many parameters reaching full compliance following the intervention. However, persistent deficiencies in documenting hospital file numbers and providing justification for withholding antibiotics highlight areas requiring continued attention. Ongoing audit cycles and the gradual adoption of electronic documentation systems are recommended to sustain and further strengthen these improvements.
